# Enhancing social media engagement using AI-modified background music: examining the roles of event relevance, lyric resonance, AI-singer origins, audience interpretation, emotional resonance, and social media engagement

**DOI:** 10.3389/fpsyg.2024.1267516

**Published:** 2024-04-15

**Authors:** Xiaohui Gu

**Affiliations:** Conservatory of Music, Communication University of Zhejiang, Hangzhou, China

**Keywords:** social media engagement, AI-modified background music, event relevance, lyric resonance, AI-singer origins, audience interpretation, emotional resonance

## Abstract

**Introduction:**

Drawing on the S-O-R model, this study aims to investigate the influence of three stimuli from AI-modified music (i.e., event relevance, lyric resonance, and AI-singer origins), two responses from social media content consumers (i.e., audience interpretation and emotional resonance) on the social media engagement of personalized background music modified by artificial intelligence (AI).

**Methods:**

The structural equation modeling analyses of 467 social media content consumers’ responses confirmed the role of those three stimuli and the mediating effect of audience interpretation and emotional resonance in shaping social media engagement.

**Results:**

The findings shed light on the underlying mechanisms that drive social media engagement in the context of AI-modified background music created for non-professional content creators.

**Discussion:**

The theoretical and practical implications of this study advance our understanding of social media engagement with AI-singer-originated background music and provide a basis for future investigations into this rapidly evolving phenomenon in the gig economy.

## 1 Introduction

In recent years, artificial intelligence (AI) has revolutionized the way social media content, such as music and art, is produced and consumed, generating new career opportunities in social media content creation (Suh et al., 2021; Fteiha et al., 2024). This paradigm shift not only empowers professionals and artists but also extends opportunities to ordinary digital users to engage in content creation for income generation. Digital platforms such as Facebook Live, Metaverse, TikTok, Twitter, YouTube, and WeChat have allowed non-professional content creators to share self-made, engaging and high-quality content with global audiences (Rodgers et al., 2021). Despite the burgeoning interest in leveraging AI for content creation, a fundamental question remains largely unaddressed: how does AI-modified music, as different from human-generated music, affect listeners?

The integration of AI in music dates back to the mid-1960s, with early research focusing on music as a cognitive process and algorithmic composition (Berz and Bowman, 1995). The integration of AI technology may help unlock new dimensions of artistic expression and audience engagement (Zulić, 2019). AI-driven innovations such as AIVA, may redefine traditional paradigms of musical performance and composition (Zulić, 2019). Despite such remarkable development, the integration of AI in music production is still in its nascent stages, with ample room for exploration and innovation. By elucidating the nuanced interplay between AI-modified music and human perception, this study seeks to contribute to the understanding of the evolving relationship between technology and creativity in the digital age.

AI-modified music can be different from human composed music in different manners. Understanding this difference is essential for comprehending the unique value proposition of AI-modified music in the social media content creation. Indeed, the two types of music are different in their underlying mechanisms music generation and implications for audience experience. While human composers infuse their creations with personal emotions, cultural nuances, and artistic insights, AI algorithms operate based on computational models and data-driven analyses (Zulić, 2019). Consequently, music purely modified by AI may lack the depth of human expression and the contextual richness inherent in human-created compositions. Moreover, the proliferation of AI in the creative domain engenders concerns regarding the authenticity and emotional resonance of AI-generated art (Trunfio and Rossi, 2021). Therefore, alongside exploring the instrumental role of AI-modified music in social media engagement, this study seeks to critically evaluate the perceptual and emotional dimensions of AI-generated compositions vis-à-vis their human counterparts.

The evolution of social media has facilitated the rise of non-professional content creators who leverage various forms of content, including gaming, tutorials, and talk shows, to interact with their audience in real-time (Spangler, 2023). Music, as a central element in content creation, plays a significant part in shaping atmosphere, evoking emotions, and influencing audience perceptions (Kuo et al., 2013; Schäfer et al., 2013; Hudson et al., 2015). Particularly noteworthy is the prevalence of AI-powered tools, such as RIADA and Juedeck, which enable non-professional creators to customize and personalize music to align with their content (Civit et al., 2022; Dredge, 2023). These tools offer features such as tempo adjustment, mood customization, and even the creation of entirely new compositions, thereby providing creators with unprecedented flexibility in tailoring music to suit their specific needs (Alvarez et al., 2023).

Non-professional content creators in this study refer to the individuals who produce various forms of content to showcase their skills, talents, and expertise in activities such as gaming, tutorials, and talk shows in real-time, creating a sense of immediacy and interactivity with their audience through social media. Over 50% of U.S. non-professional creators have profited from their content, with over 75% starting the practice within one year (Spangler, 2023). Music often plays a critical role in creating atmosphere and arousing emotions (Kuo et al., 2013; Schäfer et al., 2013; Hudson et al., 2015), affecting creators’ perceived personality and attractiveness (Yang and Li, 2013). Songs can express individuals’ inner feelings, produce sensations, and bring audiences to various emotional states by sharing an emotional state, triggering specific memories (Schön et al., 2008). In particular, non-professional content creators often perform (e.g., dance or sing) with songs of modified lyrics to produce viral video content. For instance, 34% of the most popular non-professional content creators on TikTok attributed their success to dancing content with background music (Shutsko, 2020).

Unlike studies that focus on the power of AI on professional artists (Anantrasirichai and Bull, 2022; Kadam, 2023), this study focuses on the AI-modified social media music that non-professional content creators develop to add value to their social media content rather than selling music works. This focus makes practical significance considering the size of the gig economy, which is expected to grow from USD 455.2 billion in 2023 to USD 1,864.16 billion by 2031, according to the Business Research Insights report (2023). Music-AI developers such as Soundful and Ecrett^1^ have provided services to facilitate the creation of royalty-free music for social media content, game development, and video production. Indeed, the ability to personalize background music using AI holds immense potential for enhancing the cognitive and affective engagement and satisfaction of social media content consumers (Hwang and Oh, 2020; Gray, 2023).

The literature has captured the advantages of AI-facilitated music creation, including breaking conventional musical structures, enabling listeners to tailor music, and adapting lyrics and vocal styles to suit various cultural contexts (Civit et al., 2022; Deruty et al., 2022). As a result, non-professional non-professional content creators can modify lyrics and infuse local elements to create personalized songs in response to special events (e.g., birthdays, celebrations, and social incidents). Those modified songs can provide meaningful expression of creators’ personal experiences and foster empathy among audiences. Moreover, AI allows music consumers to perform songs using the voice and singing techniques of their favorite singers, thereby extending the original singers’ limitations due to language and cultural differences. Despite the growing popularity of personalized background music, limited attention has been given to the role of AI-modified music in increasing audience engagement in content created by non-professionals. As such, studying the effect of AI-facilitated musical tools can not only inspire creative artists to connect with audiences by experimenting with different musical elements (Grech et al., 2023) but also empower non-professional content creators to commercialize their ideas.

In this context, not much is known about the factors that influence users’ social media engagement, i.e., social media consumers’ positive cognitive, emotional and behavioral activities during or related to consumer-content interactions (Trunfio and Rossi, 2021). The first predictor of social media engagement could be event relevance, which refers to the connection between a piece of music and a specific event, with the music enhancing the emotional impact of the event or evoking extramusical feelings relevant to the event (Ibrahim, 2023). Non-professional non-professional content creators may use AI to modify existing songs to align with the mood, theme, atmosphere, and location of a specific event to attract consumer attention (Anantrasirichai and Bull, 2022). Understanding how event relevance influences social media engagement can provide insights into the importance of context-specific music in generating user interest, interactions, and discussions.

The second feature of AI-modified background music could be lyric resonance, which in this study refers to the emotional impact and connection that the lyrics of a song have on social media consumers (Deng et al., 2015). It indicates a song’s ability to evoke strong emotions, resonate with personal experiences, and create a deep sense of connection or understanding. Lyrics that evoke strong emotional responses, convey relatable narratives or express profound thoughts have the potential to spark discussions and connect content consumers on social media platforms (Mori, 2022). Exploring the impact of lyric resonance on social media engagement can shed light on the role of meaningful and relatable lyrics in enhancing social media consumer interaction and fostering a sense of community.

Third, the singer’s origins, the cultural background or heritage of singers that stimulate listeners’ emotional connection and familiarity (Hemmasi, 2020), may also affect social media engagement. AI-modified songs that mimic singers from specific regions or cultural backgrounds can stimulate online communities centered around specific musical styles or cultural interests. Examining the impact of singer origins on social media engagement can provide valuable insights into the role of cultural diversity and representation in personalized AI-generated music. As such, this study aims to examine the roles of event relevance, lyric resonance, singer origins, emotional understanding, and emotional resonance on social media engagement of personalized background music.

To investigate the three features of AI-modified music, a quantitative research study is necessary. By examining the roles of event relevance, lyric resonance, and singer origins in social media consumers’ cognitive and emotional responses, this study aims to provide valuable insights into the factors that influence social media engagement of personalized background music. The findings of this study will inform the design and development of AI-generated music systems that cater to individual preferences, enhance emotional resonance, and foster active participation and engagement on social media platforms.

## 2 Literature review and hypotheses

### 2.1 Theoretical perspective

Social media engagement studies (Carlson et al., 2018; Perez-Vega et al., 2021; Abbasi et al., 2023; Wang et al., 2023) have adopted the Stimuli-Organism-Response (S-O-R) model, which assumes that atmospheric and informational cues (S) can trigger a consumer’s cognitive and affective reactions to these cues (O) that further influence his or her behavioral responses (R) (Mehrabian and Russell, 1974; Bigne et al., 2020). In particular, researchers (Suh et al., 2021; Kang and Lou, 2022; Kadam, 2023) have increasingly recognized the role of AI in enhancing audience engagement. In the context of music, the stimuli involve lyrics, musical style and artist characteristics that affect the consumer’s perception of the music (Belfi and Kacirek, 2021; Zhang et al., 2023); the organism refers to music consumers’ personal preferences, emotional states, cultural background, and cognitive processes when processing the external stimuli (Pandita et al., 2021); response, the reactions of the organism to the stimuli, may involve engagement levels and preferences of music (Hwang and Oh, 2020).

This study follows previous studies to adopt the S-O-R model to conceptualize social media content consumers’ responses to AI-modified background music. Music consumers often resonate with music that reflects or comments on the social contexts they have experienced (Murray and Murray, 1996; DeWall et al., 2011). Particular events or experiences often form the social contexts that influence social media content creators’ choice of personalized background music (Threadgold et al., 2019) to align with the intended theme or atmosphere in the content. For instance, Nelson Mandela’s struggles for freedom and equality have been conveyed in the lyrics of ‘Guang Hui Sui Yue’, a classic song by the Hong Kong rock band Beyond^2^. The AI-moderated music can form the exposure effect where specific social events, lyrics, tunes, and singers are connected to elicit consumers’ implicit memory (e.g., paternal love), thereby capturing their attention and establishing a connection. When a song is modified with a very emotional event, it can be an effective cue to bring back the strong emotion that was felt at that moment (Arjmand et al., 2017; Schaefer, 2017). As a result, I consider the relevance of the social events reflected in the AI-moderated music as a component of stimuli.

Another important stimulus is the resonance delivered by lyrics, i.e., the degree to which the lyrics of AI-modified background music resonate with social media users’ personal experiences, emotions, or themes in their daily lives. Lyrics that evoke familiarity, connection, or emotional relevance can enhance the audience’s engagement with the music (Ruth and Schramm, 2021). Following previous studies (Ruth and Schramm, 2021), I consider resonating lyrics reflected in the AI-moderated music as a component of stimuli.

Finally, AI could imitate the voices and accents of artists from a specific area to sing the songs modified by non-professional social media creators, thereby forming stimuli to social media consumers. Indeed, individuals working and living in culturally diverse cities often bear their hometown identities and self-esteem (Whitesell et al., 2009). AI-modified songs can adopt the voices, accents, and images of singers who may also bear the same cultural and geographical backgrounds with audiences from the same areas to form cultural affinity (Yoon, 2017). In doing so, non-professional creators can foster a sense of belonging and community with their audiences. Therefore, the use of singers from a specific hometown can serve as a stimulus in social media content targeted at a specific cohort of audiences.

Audiences respond to the stimuli through cognitive and emotional processes (Juslin and Västfjäll, 2008). In particular, the cognitive process involves audiences’ understanding and interpretation of AI-moderated songs. This may involve social media users’ understanding of the lyrics, the music composition, and the overall context of the AI-modified songs, i.e., whether consumers can extract the meaning and assimilate the information that the non-professional creators are trying to deliver. It may also involve consumers’ emotional interpretation, i.e., how they perceive and understand the emotional content conveyed by the AI-modified music. Previous studies (Juslin and Västfjäll, 2008; Hill, 2013) have recognized the importance of audience interpretation for audiences to connect with musicians and highlighted the need to explore underlying mechanisms of audience response to music. Moreover, these users integrate their cultural backgrounds, life experiences, and musical preferences into the interpretation process. As such, social media content consumers may attribute specific meaning, personal relevance, and subjective understanding to the music. These individualized cognitive processes collectively influence how they perceive and make sense of the AI-moderated songs. Drawing on the above discussion, this study considers audience interpretation of AI-modified songs as an organism (O) factor.

This study follows previous scholars (Tubillejas-Andres et al., 2020; Nagano et al., 2023) to adopt music consumers’ emotional response as the response factor in the SOR model. Audiences’ organismic processes may also involve emotional resonance, i.e., the degree of emotional connection, alignment, and resonance experienced by the audience with the personalized background music (Qiu et al., 2019). Social media engagement represents the content consumers’ responses to AI-modified music. It involves interactive behaviors, including likes, comments, shares, and discussions related to personalized background music on social media platforms. It reflects the level of involvement, connection, and interest that individuals exhibit toward AI-modified music and their willingness to actively participate and engage with it in the social media environment (Threadgold et al., 2019; Kang and Lou, 2022). Therefore, we consider how the three key elements of AI-modified songs (i.e., event relevance, lyric resonance, and singer origins) influence emotional interpretation and emotional resonance (organismic processes), which in turn impact social media engagement of AI-modified background music (response).

### 2.2 Event relevance

Social media content consumers’ interpretation of musical works relies on cognitive and communicative efforts, which can be enhanced through inferences (Fraser et al., 2021). AI-modified background music provides familiar contexts where social media content consumers can understand the events, sensations, and memories that they have experienced before. AI-modified background music can be specifically tailored to different events, such as weddings, parties, and relaxation scenarios. This allows content creators to align with content consumers’ experiences or memories of previous events (Gan et al., 2020). The relevance of events delivered by AI-modified background music could also influence social media content consumers’ emotional resonance. Indeed, personalized background music allows social media content consumers to find emotional connections and overall positive emotional resonance when the music is contextually relevant to the given event. Previous studies (Morris and Boone, 1998; Juslin and Västfjäll, 2008; Kwon et al., 2022) have provided empirical evidence on the role of background music in enhancing consumers’ arousal, affect, and attention to ongoing social media content. In particular, compared to original songs, AI-modified music may stimulate a stronger emotional connection and overall positive interpretation of the AI-modified music when it is contextually closer to the given event. As such, AI-modified music can remind the audience of romantic encounters, career milestones, or personal achievements. In short, social media content consumers are more likely to interpret and emotionally resonate with personalized background music that aligns with specific events. Based on such discussion, this study predicts the following hypotheses:

H1a: Event relevance has a positive impact on audience interpretation.

H1b: Event relevance has a positive impact on audience emotional resonance.

The impact of lyrics on the audience’s emotional responses has been well documented (Anderson et al., 2003; DeWall et al., 2011; Mori and Iwanaga, 2014). Music studies (Mori and Iwanaga, 2014) have associated the lyrics with emotions, especially in songs consumed in daily life. The happy and sad lyrics could stimulate social media content consumers’ perception of the pleasant and unpleasant states and situations that the songs are expressing (Vuoskoski et al., 2012; Ruth and Schramm, 2021). With the recent introduction of AI music generators such as LyricStudio and MelodyStudio, social media content creators can easily create and modify lyrics for their music. Some scholars (Louie et al., 2020) on AI-generated music have confirmed the positive impact of AI-generated lyrics on audience satisfaction with the songs. In particular, social media content creators could take advantage of social media platforms to learn about social media content consumers’ experiences, views, and opinions on various events and then integrate those elements into their content (Nwagwu and Akintoye, 2023). As such, AI-modified background music incorporating lyrics relevant to social media content consumers’ personal experiences or preferred topics in their lives can contribute to positive interpretation and emotional resonance. This suggests that lyric resonance plays a crucial role in influencing how audiences interpret and feel about AI-modified background music. Based on the above discussion, the following hypotheses can be predicted:

H2a: Lyric resonance positively influences audience interpretation.

H2b: Lyric resonance positively influences emotional resonance.

While not much has been written about the role of singers’ hometown origins on consumers’ interpretation of AI-modified background music on social media, previous studies (Oh and Lee, 2014; Fraser et al., 2021) shed light on the role of pop stars origins and hometowns in different contexts. For instance, American celebrities touring hometowns were often met with cheers and screams from audiences who share the same hometown roots (McClain, 2010). These stars can serve as hometown brand that further stimulates the understanding of shared experiences, places, and emotions that further generate a sense of belonging (Wei et al., 2023). Now AI music tools enable a piece of specific background music to be performed by virtually any singer. This function could allow social media content creators to achieve cultural affinity with content consumers by using a singer of the same hometown origins to sing a modified song (e.g., with hometown accents). As such, social media content consumers are likely to form cultural connections and develop favorable interpretations and emotional resonances to the social media content. As a result, the following hypotheses can be predicted:

H3a: Singers’ hometown origins positively influence audience interpretation of AI-generated personalized background music.

H3b: Singers’ hometown origins positively influence the emotional resonance of audiences.

Social media engagement involves an audience’s positive cognitive and emotional activity during interactions on social media (Delbaere et al., 2021). Cognitive processing involves a social media content consumer’s degree of understanding and thought about a specific social media content or personal brand (Hollebeek, 2011). Several scholars found that social media engagement can be achieved when audiences can understand the values and meaning of the messages (Shang et al., 2017; Cheng et al., 2020). AI tools can help analyze social media content consumers’ favorite music types and predict the music elements (e.g., themes and lyrics) that are more likely to generate audience liking, commenting, and sharing. As such, social media content creators can integrate those elements into their social media content to help content consumers better understand the tones, attitudes, and origins of and form and interpret the music in a meaningful and engaging manner, leading to increased social media interactions and engagement metrics such as likes, comments, and shares.

Likewise, emotional resonance achieved through AI-modified music could grab users’ attention and evoke user engagement (Shang et al., 2017; Schreiner and Riedl, 2019). When AI-modified background music is able to resonate with audience emotions, audiences are likely to develop positive sentiments and thus foster meaningful interactions with social media content consumers. This is particularly true when content consumers find a strong emotional connection to the AI-modified music content: the sense of community will encourage these audiences to join discussions and conversations. The sense of community could further encourage engagement as users actively participate in discussions, conversations, and reposts (Tafesse and Wien, 2018). As a result, the following hypotheses can be predicted:

H4a: Audience interpretation is positively associated with audience social media engagement related to AI-modified background music.

H4b: Audience emotional resonance is positively associated with audience social media engagement related to AI-modified background music.

In addition to its direct impact, positive audience interpretation from social media consumers could also be the relationship between the three stimuli from AI-modified background music and social media engagement. The special meaning achieved by relating to specific events, using modified lyrics, and adopting singers of specific origins could provide profuse information and facilitate effective communication between social media content creators and content consumers, which has been found to positively influence social media engagement (Osterholt, 2021; Nwagwu and Akintoye, 2023). Hence, this study further predicts the following mediating effect of audience interpretation:

H5a: Audience interpretation positively mediates the relationship between event relevance and social media engagement related to AI-modified background music.

H6a: Audience interpretation positively mediates the relationship between lyric resonance and social media engagement related to AI-modified background music.

H7a: Audience interpretation positively mediates the relationship between AI-singer origins and social media engagement related to AI-modified background music.

Moreover, Emotional resonance felt by social media consumers could also mediate the relationship between the three stimuli from AI-modified background music and social media engagement. Several scholars (Morris and Boone, 1998; Juslin and Västfjäll, 2008) have recognized the role of music in evoking emotions through mechanisms beyond music. For instance, the contagious influence of other content consumers from social media can be achieved when exciting events, touching lyrics, and popular singers are integrated into a piece of background music to provoke strong emotions among them. Therefore, this study predicts the following mediating effect of emotional resonance:

H5b: Emotional resonance positively mediates the relationship between event relevance and social media engagement related to AI-modified background music.

H6b: Emotional resonance positively mediates the relationship between lyric resonance and social media engagement related to AI-modified background music.

H7b: Emotional resonance positively mediates the relationship between AI-singer origins and social media engagement related to AI-modified background music.

**FIGURE 1:**
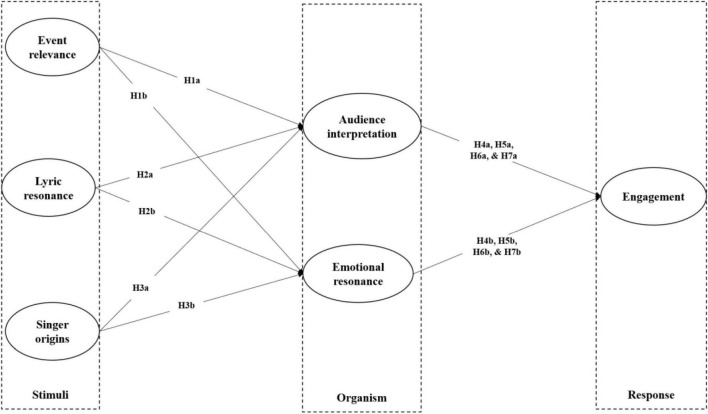
Conceptual framework.

The above hypotheses can form the following conceptual framework (see Figure 1).

## 3 Materials and methods

### 3.1 Data collection procedure

The data used in this study were collected through WENJUANXING, a popular online survey platform in southeastern China. This study distributed the questionnaire after explaining the research purpose to the respondents. Participants were informed that they could leave without repercussions and that their data would only be used for publication and not be shared with other organizations. By confirming this information, this study attempted to ascertain that they shared their thoughts. After excluding invalid responses, 467 valid samples remained from the 500 questionnaires collected. Among the 467 participants, 238 were male (51%) and 229 females (49%).

The highest proportion of respondents aged 26 to 30 is 31%, followed by the 19–25 years old group (28.3%). This result indicates a significant presence of young adults in our study. Furthermore, 74% of respondents hold bachelor’s degrees, suggesting a predominantly educated participant pool. This demographic distribution with previous research highlighting university students and young adult graduates as key demographics in studies regarding music consumption and technology adoption (Karim et al., 2020; Li, 2022). According to Li (2022), individuals in university and young adult life stages demonstrate a heightened interest in music idols and celebrities. This interest often extends to their engagement with novel technologies, as highlighted by Karim et al. (2020), who found that university students and young adult graduates are frequently early adopters of new technologies, such as AI-mediated music platforms.

The selection of university students and young adult graduates as the primary demographic for this study is well-founded given their significance in both music consumption and technology adoption behaviors. Previous studies have underscored the relevance of this demographic group in understanding trends in digital music consumption and the adoption of innovative music technologies. For instance, Borja et al. (2015) explored the preferences and behaviors of international university students regarding music streaming platform, highlighting their role as early adopters in the digital music landscape. Likewise, Cha et al. (2020) investigated the impact of technological advancements on the pop music consumption of young adults, reinforcing the importance of studying this demographic in the context of music and technology.

Moreover, the demographic profile of university students often includes a diverse range of cultural backgrounds, socioeconomic statuses, and technological proficiencies (Simon and Ainsworth, 2012), thus enriching the breadth and depth of our study findings. Furthermore, the inclusion of young adult graduates in our study extends our scope beyond the confines of universities, offering insights into the post-graduation phase when individuals are navigating career transitions and establishing their identities as consumers in the digital marketplace.

Moreover, 38.5% of respondents indicated employment in private enterprises, followed by the 21.4% of self-employed respondents, 18.8% of students, and 14.3% of government employees (civil servants). Such information reflects a diverse occupational background among participants. Table 1 shows the demography statistics of the respondents.

**TABLE 1:** Demography information.

Demography		Frequency	Percentage
Gender	Male	238	51.0%
	Female	229	49.0%
Age	19–25 years old	132	28.3%
	26–30 years old	145	31.0%
	31–35 years old	110	23.6%
	36–40 years old	46	9.9%
	41 years old and above	34	7.3%
Edu	College degree or below	47	10.1%
	Undergraduate degree	349	74.7%
	Master’s degree or above	71	15.2%
Occupation	Student	88	18.8%
	Civil servant	67	14.3%
	Self-employed employees	100	21.4%
	Private enterprise employees	180	38.5%
	Others	32	6.9%

In short, the convergence of empirical evidence from previous research, alongside the detailed demographic profile of our sample, strengthens the credibility and relevance of our study findings in advancing scholarly understanding and practical applications in the field of digital music and consumer behavior.

### 3.2 Measures

To ensure the reliability and validity of the questionnaire, all items used to measure the variables in this study were adapted from previously published scales. As this study collected data from China, the questionnaire was administered through a translation process in both directions. Following Brislin (1970), two researchers independently translated the questionnaire from English to Mandarin and back to English. To ensure the accuracy of the translation, this study consulted with other researchers. All measures of this study were applied using a 5-point Likert scale (1: strongly disagree; 5: strongly agree), except AI-singer origins, which were measured by a binary item adopted from Zhu et al. (2022), a dummy variable that equals 1 if the singers are from the AI technology, and 0 otherwise. The measurement of event relevance was adopted from Wang (2019), which includes fifteen items, such as “The background music connects to the details of events relevant to me.” The scale of lyric resonance adopted the measurement items proposed by Lee et al. (2021). The measurements of audience interpretation were from Alsaad et al. (2023), which include two items. Emotional resonance was measured by fifteen items adopted from Toivo et al. (2023). The measurements of social media engagement were derived from Alsaad et al. (2023) and consisted of ten items divided into three subconstructs: consumption, contribution, and creation. The detailed measurement items are attached in Supplementary Appendix.

### 3.3 Analysis

SPSS 25.0 and AMOS 24.0 were utilized for statistical analysis. Initially, descriptive statistics, CMB (common method bias), reliability analysis and correlation analysis were conducted using SPSS. Then, AMOS was used to conduct a confirmatory factor analysis (CFA) to evaluate measurement model. To assess model fit, this study employed conventional indexes and some of the most commonly used standards in the literature (CMIN/DF < 3, CFI > 0.90, RMSEA, and SRMR < 0.08). Reliability testing uses Cronbach alpha and composite reliability to test the reliability of the scale variables. This study employed structural equation modeling (SEM) with maximum likelihood estimation to evaluate each hypothesis. Moreover, the bootstrap method with a sample size of 5,000 was utilized to test the mediation effects. By controlling the effects of measurement error, this method can reduce bias in estimating estimates in a mediation analysis (Ledgerwood and Shrout, 2011).

## 4 Results

### 4.1 Common method bias

Because the data were self-reported by live stream users, there might be the issue of common method bias (CMV). To examine this issue, this study used Harman’s single-factor method for CMB testing. This study included all the items in the exploratory factor analysis (EFA). Through the analysis results, it can be seen that the variance explanation rate of the first factor is 37.801%, less than 40%, indicating that there is no serious problem of common method bias in this study.

### 4.2 Reliability and validity

Cronbach’s alpha and composite reliability (CR) were utilized to assess the variables’ internal consistency and dependability. The results indicate that the value of each variable exceeds the recommended threshold of 0.7, and the reliability is good. All variable factor loading coefficients and average variance extraction (AVE) are greater than 0.5, indicating convergence validity (see Table 2). The model fit of the measurement model in this study is CMIN/DF = 2.49, CFI = 0.928, SRMR = 0.045, and RMSEA = 0.057, indicating that the structural validity of this study is good. The results for comparing the correlation coefficient and square roots of AVEs of every variable indicate that discriminant validity is good (see Table 3).

**TABLE 2:** Reliability and validity.

Construct	Item	Factor loading	CR	AVE
Social media engagement	CONS	0.728	0.788	0.555
	CONT	0.828		
	CRE	0.670		
Event relevance	EVE1	0.828	0.972	0.702
	EVE2	0.892		
	EVE3	0.837		
	EVE4	0.852		
	EVE5	0.833		
	EVE6	0.818		
	EVE7	0.861		
	EVE8	0.813		
	EVE9	0.815		
	EVE10	0.756		
	EVE11	0.781		
	EVE12	0.893		
	EVE13	0.798		
	EVE14	0.873		
	EVE15	0.905		
Lyric resonance	LYR1	0.787	0.902	0.699
	LYR2	0.839		
	LYR3	0.771		
	LYR4	0.938		
Audience interpretation	AUDI1	0.865	0.829	0.709
	AUDI2	0.818		
Emotional resonance	EMO1	0.873	0.973	0.708
	EMO2	0.826		
	EMO3	0.898		
	EMO4	0.922		
	EMO5	0.864		
	EMO6	0.838		
	EMO7	0.828		
	EMO8	0.815		
	EMO9	0.814		
	EMO10	0.821		
	EMO11	0.807		
	EMO12	0.825		
	EMO13	0.771		
	EMO14	0.868		
	EMO15	0.835		
Consumption	CONS1	0.862	0.911	0.720
	CONS2	0.820		
	CONS3	0.896		
	CONS4	0.813		
Contribution	CONT1	0.808	0.854	0.662
	CONT2	0.866		
	CONT3	0.764		
Creation	CRE1	0.775	0.839	0.634
	CRE2	0.784		
	CRE3	0.829		

**TABLE 3:** Discriminant validity and correlations.

Variable	Mean	SD	1	2	3	4	5	6	7	8	9	10
Gender	1.49	0.50	–									
Age	2.37	1.20	0.013	–								
Edu	2.05	0.50	−0.049	−0.060	–							
Occupation	3.00	1.25	0.019	−0.028	0.038	–						
Event relevance	3.56	0.98	−0.023	0.042	0.029	0.018	0.838					
Lyric resonance	3.81	1.05	−0.004	0.040	−0.002	0.033	0.446**	0.836				
AI-singer origins	0.55	0.50	−0.035	0.030	0.015	−0.081	0.126**	0.176**	–			
Audience interpretation	3.81	0.93	0.020	−0.038	−0.011	0.011	0.313**	0.318**	0.194**	0.842		
Emotional resonance	3.58	0.98	0.072	0.071	0.048	0.051	0.330**	0.397**	0.292**	0.371**	0.841	
Social media engagement	3.70	0.84	−0.009	0.012	0.017	−0.003	0.406**	0.388**	0.189**	0.358**	0.347**	0.745

**p < 0.01; Square roots of AVEs on diagonal.

### 4.3 Structural equation model analysis

A structural equation model (SEM) was conducted to test hypotheses by using AMOS. The initial model containing only the predictor, mediator, and outcome shows an excellent fit to the data (CMIN/DF = 2.52, CFI = 0.923, SRMR = 0.065, and RMSEA = 0.057). Table 4 displays the model’s path coefficients. Event relevance (β = 0.163, *p* < 0.05), lyric resonance (β = 0.309, *p* < 0.05), and AI-singer origins (β = 0.218, *p* < 0.05) have significant positive effects on emotional resonance; event relevance (β = 0.225, *p* < 0.05), lyric resonance (β = 0.250, *p* < 0.05), and AI-singer origins (β = 0.159, *p* < 0.05) have significant positive impacts on audience interpretation; emotional resonance (β = 0.280, *p* < 0.05) and audience interpretation (β = 0.406, *p* < 0.05) have significant positive impacts on social media engagement. This evidence supports H1a, H2a, H3a, H4a, H1b, H2b, H3b, and H4b.

**TABLE 4:** Results of hypotheses testing.

Path	Std. estimate	S.E.	C.R.	*P*	Support or not
Emotional resonance <— Event relevance	0.163	0.047	3.360	***	Supported
Audience interpretation <— Event relevance	0.225	0.044	4.135	***	Supported
Emotional resonance <— Lyric resonance	0.309	0.057	6.003	***	Supported
Audience interpretation <— Lyric resonance	0.250	0.053	4.414	***	Supported
Emotional resonance <— AI-singer origins	0.218	0.086	5.183	***	Supported
Audience interpretation <— AI-singer origins	0.159	0.081	3.400	***	Supported
Social media engagement <— Emotional resonance	0.280	0.034	5.221	***	Supported
Social media engagement <— Audience interpretation	0.406	0.047	6.473	***	Supported

***p < 0.001.

### 4.4 Mediating effects testing

This study further examined the serial mediation effects of emotional resonance and audience interpretation on the association between event relevance, lyric resonance, AI-singer origins and social media engagement. This study examined the mediation effects using bootstrapping method in AMOS. The results are shown in Table 5. The results showed that the significant indirect effect of event relevance on social media engagement via emotional resonance is 0.046, 95% CI (confidence interval) = [0.015, 0.094]. The significant indirect effect of event relevance on social media engagement via audience interpretation is 0.091, 95% CI = [0.037, 0.163]. The significant indirect effect of lyric resonance on social media engagement via emotional resonance is 0.086, 95% CI = [0.045, 0.142]. The significant indirect effect of lyric resonance on social media engagement via audience interpretation is 0.101, 95% CI = [0.046, 0.179]. The significant indirect effect of AI-singer origins on social media engagement via emotional resonance is 0.061, 95% CI = [0.031, 0.101]. The significant indirect effect of AI-singer origins on social media engagement via audience interpretation is 0.065, 95% CI = [0.027, 0.109]. Thus, H5a, H6a, H7a, H5b, H6b, and H7b are supported.

**TABLE 5:** Mediating effects.

Path	Estimate	Lower	Upper	*P*
Event relevance-Emotional resonance-Social media engagement	0.046	0.015	0.094	0.001
Event relevance-Audience interpretation-Social media engagement	0.091	0.037	0.163	0.001
Lyric resonance-Emotional resonance-Social media engagement	0.086	0.045	0.142	0.000
Lyric resonance-Audience interpretation-Social media engagement	0.101	0.046	0.179	0.000
AI-singer origins-Emotional resonance-Social media engagement	0.061	0.031	0.101	0.000
AI-singer origins-Audience interpretation-Social media engagement	0.065	0.027	0.109	0.001

## 5 Discussion

Drawing on the growing interest from researchers and practitioners (Civit et al., 2022), this study explores how AI enables non-professional content creators to create and modify music to add value to their videos, games, and other content (Dredge, 2023). The structural equation modeling analysis using AMOS 24 provided empirical support to the hypothesized conceptual framework. Analysis results indicate that the model adequately represents the relationships among the variables under investigation. In doing so, this study makes several contributions.

First, the focus on AI-modified music by non-professional content creators adds to the music studies that focus on AI-facilitation in music creation among professional artists (Anantrasirichai and Bull, 2022). More importantly, this study sheds light on the growing body of literature on the gig economy (Waldkirch et al., 2021), where ordinary individuals such as non-professionals are also able to create social media content to generate income. Our findings suggest that non-professional content creatorscan use AI to tailor songs sung by singers with cultural affinity with modified lyrics to elicit content consumers’ experiences or memories regarding important social events. In doing so, we contribute to the AI-music literature by unraveling the key elements that AI enables those creators to develop to achieve consumer engagement.

Second, my findings extend the S-O-R model by contextualizing the three stimuli (e.g., event relevance, lyric resonance, and AI-singer origins) from AI-modified music; content consumers’ cognitive (i.e., audience interpretation) and emotional (emotional resonance) processes (O) that further affect their social media engagement (R) behavior. Specifically, the impact of event relevance, lyric resonance and AI-singer origins on social media content consumers’ interpretation and emotional resonance concurred with previous studies on the role of background music in arousing content consumers’ emotional changes (Juslin and Västfjäll, 2008). In particular, I elaborated on how AI-modified background music can stimulate content consumers’ emotions by reminding them of previous events (e.g., milestones and achievements) that are important to their individual lives and how cultural affinity is achieved to stimulate content consumers’ sense of belonging.

Third, I elaborate on the role of social media content consumers’ cognitive and emotional processing in influencing social media engagement. In particular, I proved the serial mediation effects of emotional resonance and audience interpretation on the relationship between event relevance, lyric resonance, AI-singer origins, and social media engagement. The integration of these research findings with existing literature is noteworthy. Prior studies have emphasized the role of cognitive understanding and emotional resonance in shaping consumers’ responses to various media content (Qiu et al., 2019; Delbaere et al., 2021). This study contributes to this body of knowledge by examining these mechanisms in the context of AI-modified music. In particular, it adds empirical evidence regarding how social media content consumers’ interpretations of lyrical content and emotional connections with music play a pivotal role in fostering social engagement with AI-generated background music.

The practical implications of research findings are twofold. First, non-professional, non-professional content creators and digital platforms can leverage the identified factors of event relevance, lyric resonance, and AI-singer origins to enhance social media content consumers’ cognitive understanding and emotional experiences of social media content. This can be achieved by adopting AI to learn the popular events, themes, and emotions shared by different categories of social media content consumers. Realizing and integrating those elements into background music can lead to increased social media engagement, facilitating a wider reach and greater impact. Second, understanding the mediating effects of audience interpretation and emotional resonance is valuable guidance for non-professional content creators to adopt effective strategies for attracting social media consumers. By encouraging audience understanding and eliciting emotional responses to the background music, non-professional content creators can foster a more engaged and loyal content consumer base.

Despite the aforementioned contributions and insights, this study is subject to some limitations. First, this study focused on a specific genre of music, i.e., the AI-modified singer model. While addressing the research gaps, it may also limit the generalizability of the findings to AI-generated music genres, especially those produced by professional musicians. Second, the study adopted self-reported measures for audience interpretation, emotional resonance, and social media engagement, which may be subject to response biases. Future studies could consider integrating physiological measures or behavioral observations to complement self-reported data. Third, findings of this study are not causal due to the chosen research design, which primarily relies on survey data and structural equation modeling. While these methods provide valuable insights into associations between variables, they do not allow for manipulation of possible confounding variables that may influence the observed relationships. Future studies can adopt controlled experiments to confidently make causal claims. Fourth, this study only focused on social media engagement as a dependent variable. Future studies could develop and examine other potential dimensions of social media engagement with AI-modified music. Expanding the scope of engagement measures could provide a more comprehensive understanding of social media content consumers’ responses to AI-modified music.

## 6 Conclusion

This study sheds light on the underlying mechanisms that drive social media engagement in the context of AI-modified background music. By investigating the roles of event relevance, lyric resonance, AI-singer origins, audience interpretation, and emotional resonance, this study provide valuable insights for researchers and practitioners in the social media and digital music business. The theoretical contributions and practical implications of this study advance our understanding of social media engagement with AI-singer-originated background music and provide a basis for future investigations in this rapidly evolving domain.

## Data availability statement

The raw data supporting the conclusions of this article will be made available by the author, without undue reservation.

## Ethics statement

The studies involving humans were approved by the Research Department, Communication University of Zhejiang. The studies were conducted in accordance with the local legislation and institutional requirements. The participants provided their written informed consent to participate in this study.

## Author contributions

XG: Writing – review and editing, Writing – original draft, Methodology, Investigation, Formal analysis, Data curation.

## Footnotes

1
https://www.makeuseof.com/free-ai-music-generators-create-unique-songs/

2
https://www.lyrics.com/lyric/28688428/Beyond/Guang+Hui+Sui+Yue
