# Supplementary material for: Enhancing social media engagement using AI-modified background music: examining the roles of event relevance, lyric resonance, AI-singer origins, audience interpretation, emotional resonance, and social media engagement
Source: Front Psychol. 2024 Apr 15;15:1267516. doi: 10.3389/fpsyg.2024.1267516 (PMC11057495; doi:10.3389/fpsyg.2024.1267516)
Supplement: Supplementary file 1 [file Data_Sheet_1.docx]

Appendix : Measurements (English/Chinese)

**Event relevance 事件相关性**

1. I prefer AI-modified music that resonates with the theme or atmosphere of the event in the social media. 当AI修改过的音乐能反映出社交媒体中涉及的主题或氛围时，我会非常喜欢。
2. Listening to AI-modified music becomes more enjoyable for me when it aligns with the occasion or theme in the social media. 当AI修改过的音乐与社交媒体中涉及的场合或主题相符时，我会感到更加愉快。
3. AI-modified music is most satisfying when it captures a specific event in the social media that holds significance for me. AI修改过的音乐在捕捉对我具有重要意义的社交媒体中涉及的事件时，听起来更有满足感。
4. Discussing AI-modified music with others enhances my appreciation for its relevance to specific events or occasions in the social media. 与他人讨论AI修改过的音乐有助于我对社交媒体中涉及的事件或场合的欣赏。
5. Sharing my experiences with AI-modified music allows me to connect with others by discussing its relevance to particular events in the social media. 通过分享我对AI修改过的音乐的体验，我能够通过与他人一起讨论社交媒体中涉及的事件而建立联系。
6. I am often drawn to AI-modified music that enhances the atmosphere of specific events in the social media. 能够增强社交媒体中涉及事件的氛围的AI修改过的音乐往往能够吸引我。
7. AI-modified music brings me closer to the event in the social media. AI修改过的音乐能够拉近我与社交媒体中涉及的事件的联系。
8. I feel pleased to read social media content with AI-modified music as background. 我喜欢阅读以带有AI修改音乐为背景的社交媒体内容。
9. Even if the quality is not exceptional, I derive pleasure from AI-modified background in the social media. 即使社交媒体的内容质量不是特别出色，我也能通过欣赏AI修改过的背景音乐中获得快乐。
10. My favorite AI-modified music is that that I can share with friends and loved ones during memorable events in the social media. 我最喜欢的AI修改过的音乐是那些我可以在社交媒体中的难忘事件中与朋友和亲人分享的音乐。
11. I prioritize listening to AI-modified music that enhances the ambiance of events in the social media, even if it is not of exceptional quality. 我更重视那些能增强社交媒体中事件氛围的AI修改过的音乐，即使它们的质量并不特别出色。
12. AI-modified music has the ability to strengthen bonds, particularly when it resonates with specific events in the social media. AI修改过的音乐能增强人们之间的联系，尤其是当它与社交媒体中涉及的特定事件产生共鸣时。
13. Listening to AI-modified music alone does not detract from its relevance to events and occasions in the social media for me. 对我来说，即使独自聆听AI修改过音乐，也不会削弱它与社交媒体涉及的事件和场合的相关性。
14. Sometimes I listen to the popular AI-modified music that shared by others due to its relevance to specific events in the social media. 我有时候会聆听其他人在社交媒体上分享与某些事件相关的AI修改过的流行音乐。
15. I enjoy listening to AI-modified music in the presence of others, especially when we share similar memories and experiences mentioned in the music. 我喜欢与他人共同聆听AI修改音乐，尤其是当我们对音乐中的内容具有相似记忆和经历时。

**Lyric resonance 歌词共鸣**

1. The lyrics of AI-modified music in the social media resonate deeply with me and provide a source of healing and comfort. 社交媒体上的AI修改音乐的歌词深深地融入了我的心灵，并成为了治愈和安慰的源泉。
2. Every single line of lyrics of AI-modified music in the social media is meaningful to me. 在社交媒体上的AI修改过的音乐的每一句歌词对我来说都是有意义的。
3. The lyrics of AI-modified music in the social media has helped me develop a greater sense of self-love and self-respect. 在社交媒体上的AI修改音乐的歌词帮助我培养了更加强烈的自爱和自尊。
4. I find solace and comfort in the lyrics of AI-modified music in the social media, and I can easily relate to the emotions expressed. 我在社交媒体上的AI修改音乐的歌词中找到了慰藉和舒适，并且我能够轻松地与所表达的情感产生共鸣。

**AI-singer origins AI歌手出生地**

1. The singers in my favorite AI-modified music in the social media come from the same city of birth (0: Yes, 1: No) 我在社交媒体上喜欢的AI修改音乐中的歌手来自同一个出生城市（0：是，1：否）

**Audience interpretation 观众解读：**

1. By listening to the AI-modified music, I can understand the meaning of the events or experiences in social media to me. 通过听AI修改过的音乐，我能够理解社交媒体上所涉及的事件或经历对于我的意义。
2. AI-modified music signifies my understanding of the events or experiences mentioned in the social media. AI修改过的音乐代表了我对社交媒体中所提到的事件或经历的理解。
3. AI-modified music helps to build a shared sense of participation in the events or experiences. AI修改过的音乐有助于我建立对事件或经历的共同参与感。

**Emotional resonance 情感共鸣：**

1. I experience more emotional depth when listening to AI-modified music compared to original songs. 当我听AI修改音乐时，我会比听原创歌曲更深地感受到情感的深度。
2. When I feel really upset, I tend to listen AI-modified music more than original songs. 当我感到非常沮丧时，我倾向于听AI修改过音乐，而不是原创歌曲。
3. Angry words have a greater impact on my emotions when sung in my native language than the language in original songs. 用我的母语唱出来的表达愤怒歌曲对我的情绪影响比原创歌曲中的语言影响更大。
4. I find it easier to understand the feelings in AI-modified music compared to original songs. 相比原创歌曲，我觉得在AI修改音乐中更容易理解情感。
5. I find it easier to feel the sorrowful emotions in AI-modified music compared to original songs. 相比原创歌曲，我觉得在AI修改音乐中更容易感受到悲伤的情绪。
6. The equivalent of “I love you” has more weight in my native language than the language in original songs. “我爱你”这样的表达通过我的母语唱出来中比原创歌曲语言唱出来更有份量。
7. I feel like there is more of an emotional distance when I listen original songs than AI-modified music. 当我听原创歌曲时，我觉得情感距离要比听AI修改音乐时要远一些。
8. Sad lyrics have a stronger emotional impact on me when conveyed in in my native language than the language in original songs. 相比原创歌曲中的语言，悲伤的歌词以我的母语表达时对我的情感影响更强。
9. I derive more pleasure from listening to AI-modified music than original songs. 我从听AI修改音乐中得到的愉悦感比听原创歌曲更多。
10. Sad songs are more likely to evoke tears when listened to in AI-modified versions rather than original ones. 相比原创歌曲而言，悲伤的歌曲在AI修改版本中更容易让我落泪。
11. The lyrics that carry emotional weight are better sung in my native language than the language in original songs. 承载情感的歌词在我的母语中唱的时候比原创歌曲所用语言唱出来达到的效果更好。
12. Stories in the lyrics have more effect on my emotions when expressed my native language than the language in original songs. 相比原创歌曲而言，歌词中的故事用我的母语表达时对我的情感影响更大。
13. I form emotional connections better with other audiences who share the same native language than those sharing the language in original songs. 我与那些使用相同母语的观众更容易建立情感联系，而与那些使用原创歌曲语言的观众之间建立情感联系则不那么容易。
14. I have a better sense of other audiences’ thoughts and feelings communicating about the AI-modified music in my native language than doing it in the language in original songs. 相比原创歌曲所用语言，我用我的母语交流关于AI修改音乐的想法和感受时，更好地理解其他观众的想法和感受。
15. Romantic songs evoke more intense emotions when presented in AI-modified versions in the social media compared to original ones. 用AI修改过的浪漫的歌曲在社交媒体上以比原创歌曲更能引发强烈的情感。

**Social media engagement 社交媒体参与**

1. I actively engage with posts featuring AI-modified music as background on social media. 我积极参与回复社交媒体上以AI修改音乐为背景的帖子。
2. I follow fan pages showcasing AI-modified music as background on social network sites. 我关注以AI修改音乐为背景的社交网络网页。
3. I regularly read blogs featuring AI-modified music as background. 我定期阅读以AI修改音乐为背景的博客。
4. I am a follower of AI-modified music profiles on social network users. 我会关注以AI修改的音乐为背景的社交网络账号。
5. I “Like” posts featuring AI-modified music as background. 我会给以AI修改音乐为背景的帖子点赞。
6. I actively participate in discussions on posts featuring AI-modified music as background by leaving comments. 我积极参与讨论以AI修改音乐为背景的帖子并留言。
7. I share social media posts featuring AI-modified music as background with my network. 我会分享以AI修改的音乐为背景的社交媒体帖子。
8. I contribute to online forums by creating posts featuring AI-modified music as background. 我会在线论坛上创建以AI修改音乐为背景的帖子。
9. I take the initiative to share posts featuring AI-modified music as background on social network sites. 我会主动在社交网络网站上分享以AI修改音乐为背景的帖子。
10. I write reviews to social media content with AI-modified music as background. 我会撰写关于以AI修改音乐为背景的社交媒体内容的评论。
